# Advanced Plasma-Modified Textile Polymer Materials for Building Energy Retrofit Technologies

**DOI:** 10.3390/polym18111395

**Published:** 2026-06-04

**Authors:** Musaddaq Azeem, Nesrine Amor, Muhammad Kashif, Muhammad Tayyab Noman

**Affiliations:** 1 Green Energy and EPC Services, 123b Barkby Road, Leicester LE4 9LG, UK; 2 3D Technology Department, Technical University of Liberec, Studentská 1402/2, 461 17 Liberec, Czech Republic

**Keywords:** buildings, plasma treatment, retrofitting, smart shading systems, textile polymers

## Abstract

Buildings account for a significant share of global energy consumption and carbon emissions, creating an urgent need for advanced energy retrofit technologies. This review critically examines the role of plasma-modified textile polymer materials in improving the energy efficiency and durability of building retrofit systems. Various textile polymers, including polyester (polyethylene terephthalate, PET), polypropylene (PP), polytetrafluoroethylene (PTFE), polyamide (PA), and fiber-reinforced composites, are evaluated in relation to plasma surface engineering approaches, including atmospheric plasma, dielectric barrier discharge (DBD), and plasma jet treatment. Reported studies demonstrate that plasma treatment significantly alters surface morphology and chemistry, resulting in increased surface roughness, enhanced wettability, improved coating adhesion, and superior hydrophobic behavior. Water contact angles increased from approximately 70° to 145° depending on polymer type and plasma conditions, while reflective coating performance improved with solar reflectance enhancements of approximately 10–15%. Plasma-treated reflective roofing and shading textiles also showed reductions in building cooling energy demand of approximately 18–25% and roof temperature decreases of 10–15 °C. Furthermore, plasma-induced surface activation improved durability, ultraviolet (UV) resistance, and weather stability of textile membranes used in facade and roofing applications. The review also discusses industrial challenges related to scalability, plasma aging effects, energy consumption, and long-term performance. Plasma-modified systems demonstrate strong potential for multifunctional, lightweight, and sustainable building envelope technologies for future energy-efficient construction.

## 1. Introduction

Buildings account for a large share of global energy consumption and play a significant role in total energy consumption and greenhouse gas emissions. The growing urban population, industrial development and increasing energy demand have made the construction sector one of the main factors of the global energy crisis [1]. For this reason, the development of efficient energy consumption and sustainable building systems has become an important research topic in today’s era. In recent decades, various technologies and materials have been introduced to improve the energy performance of buildings, in which building energy retrofit and advanced surface engineering methods are playing an important role [2,3]. This review presents an overview of the plasma surface modification of textile-based polymer materials and their potential for energy recovery of buildings. According to the International Energy Agency, buildings consume approximately 30–40% of the world’s energy and are also a significant contributor to carbon dioxide emissions [4]. A large portion of this energy is spent on heating and cooling systems, lighting, and other electrical equipment in buildings. The growing urban population and the expansion of urban areas are expected to further increase the energy demand of buildings in the coming years [5]. This increasing energy consumption is also having a profound impact on the environment, including greenhouse gas emissions, air pollution, and global warming [6]. To solve this problem, research is ongoing around the world on sustainable building systems, energy efficiency, and the use of renewable energy. Various methods are being used to improve the energy efficiency of buildings, including better insulation, modern building materials, and energy-efficient designs [7]. Despite these measures, a large number of existing buildings do not meet energy efficiency standards. Therefore, upgrading old buildings with modern technologies has emerged as an important strategy, called building energy retrofit. Since most of the buildings in the world were built several decades ago, they do not have modern energy standards [8]. In this situation, retrofit technologies play an important role in reducing energy waste and improving the performance of buildings. Various measures can be taken through building retrofit, e.g., better thermal insulation, energy-efficient windows, modern facade systems and reflective roofs. These measures can better control the internal temperature of the building, which reduces the dependence on heating and cooling systems.

In recent years, technical textile materials have also been included in building retrofit systems because of their lightweight, high-strength and flexible properties [9,10]. These materials can be used to improve the external structures, facade systems and shading systems of buildings, which significantly reduces energy consumption [11]. Technical textiles are textile materials that are used for industrial and technical purposes, rather than for traditional clothing or decoration. The use of technical textiles in the architectural and construction sector is rapidly increasing [12,13]. However, additional surface modifications are often required to improve the surface properties of these materials to further improve their performance. Plasma surface modification is a modern and environmentally friendly method used to modify the surface properties of polymer materials [14]. In this method, a gas is ionized by electrical energy to produce a plasma that induces various chemical and physical changes on the surface of the material. Plasma treatment creates active chemical groups on the surface of polymer materials, which increases the surface energy and adhesion. This results in improved adhesion of coatings and increased surface performance of the material [15]. Various properties, e.g., hydrophobicity or hydrophilicity, self-cleaning, UV resistance, and thermal reflectance, can be achieved through plasma treatment. These properties make plasma-modified systems more effective in various energy retrofit systems of buildings. Furthermore, plasma technology uses less energy and fewer chemicals than traditional chemical finishing, making this method considered more sustainable and environmentally friendly [16].

Although several review studies have discussed plasma surface modification of polymeric and textile materials, most existing reviews primarily focus on textile finishing processes, biomedical applications, surface wettability, or general plasma engineering mechanisms [17]. Similarly, previous studies on building energy retrofit technologies mainly emphasize conventional insulation materials, facade engineering, and energy-efficient building systems, with limited attention given to advanced textile polymer materials. To date, a comprehensive review specifically addressing the integration of plasma-modified textile polymer materials into building energy retrofit applications remains limited. This review differs from previous work by critically connecting plasma surface engineering, textile polymer functionalization, and energy-efficient architectural applications within a unified framework. In particular, this study focuses on how plasma-induced surface modifications influence hydrophobicity, coating adhesion, thermal reflectivity, UV resistance, durability, and self-cleaning behavior of textile polymers used in reflective roofing systems, facade retrofits, smart shading systems, and thermal insulation membranes. Furthermore, this review highlights industrial scalability challenges, long-term durability considerations, and future opportunities involving smart textiles, nanotechnology, and renewable energy integration for sustainable building envelope technologies.

## 2. Literature Review

**Figure 1:**
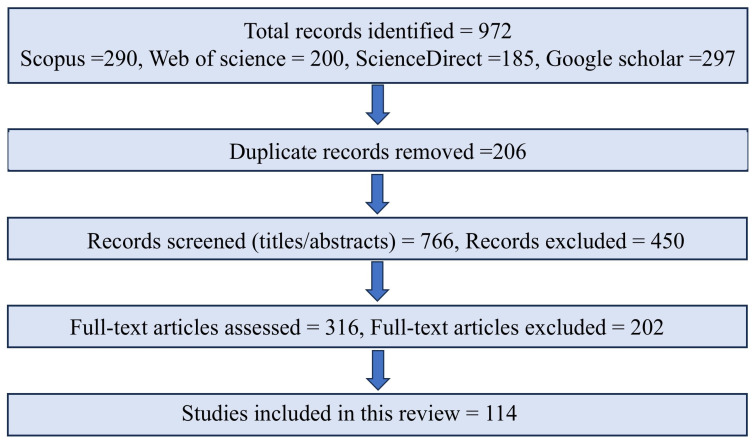
The selection of literature from various scientific databases.

This systematic process of literature selection is presented in the form of a flow diagram in Figure 1, which clearly shows the different steps of study selection. The inclusion and exclusion criteria were defined to ensure the relevance and quality of the selected literature. Studies were included if they investigated plasma surface modification of textile polymer materials; reported surface, functional, thermal, or durability-related properties relevant to architectural, construction, or energy retrofit applications; involved polymer-based textiles; and were published in peer-reviewed journals in the English language. Studies focusing exclusively on biomedical, electronic, or non-textile plasma applications without relevance to building or architectural systems were excluded. Articles lacking sufficient technical detail, duplicate publications, non-English documents, editorials, and unrelated short communications were also excluded from the review process. The literature screening process involved title and abstract evaluation followed by full-text assessment to ensure alignment with the objectives of this review. This literature review doesn’t involve any human or animal medical trial.

Several plasma-generation techniques are used for the surface modification of textile polymer materials, each offering distinct advantages in terms of operating conditions, treatment uniformity, scalability, and surface interaction mechanisms [18,19,20,21]. The selection of plasma treatment method depends on factors, e.g., polymer sensitivity, desired surface functionality, treatment area, and industrial processing requirements. Among the various approaches, atmospheric-pressure plasma, DBD, and plasma jet technologies are the most widely investigated for textile surface engineering applications. Atmospheric-pressure plasma is generated under ambient pressure conditions without the need for vacuum chambers, making it particularly attractive for large-scale industrial processing of textile materials. This technique enables continuous and roll-to-roll surface treatment, which is highly suitable for architectural textiles and membrane fabrication systems. Atmospheric plasma treatment induces rapid surface oxidation and activation through the interaction of reactive plasma species with polymer surfaces, resulting in improved surface energy, wettability, and coating adhesion [22]. In DBD system, a dielectric barrier is positioned between two electrodes to prevent arc formation and ensure stable plasma generation under atmospheric or near-atmospheric conditions. DBD plasma is particularly effective for surface activation of textile polymers because it produces reactive oxygen and nitrogen species capable of introducing polar functional groups onto polymer surfaces. The method provides precise control over treatment intensity while minimizing bulk material degradation [23]. Plasma jet systems generate a focused stream of plasma through a confined nozzle, allowing localized and highly controlled surface treatment. Unlike large-area plasma systems, plasma jets are especially suitable for selective surface engineering of geometrically complex textile structures and multilayer membrane systems. The high reactivity of plasma jets enables efficient surface activation, microstructural modification, and targeted deposition of functional coatings [24].

Numerous research studies have been conducted on the effects of plasma treatment on textile polymer materials [25,26]. In a study, Morent et al. have presented a detailed review of the surface modification of textile materials using non-thermal plasma and reported that this method is more effective and environmentally friendly than traditional chemical finishing [27]. Similarly, Montarsolo et al. have reported that plasma treatment increases the surface roughness of PET fibers, which improves adhesion to coatings [28]. The effects of plasma treatment on hydrophobic polymers (PP) have also been the subject of extensive research. Since PP has a low surface energy, it is difficult to coat or adhere to it. Plasma treatment can solve this problem because it improves wettability by creating polar groups on the surface [29]. Similarly, fluoropolymer materials (PTFE) are also considered suitable for surface modification by plasma treatment. Various studies have shown that plasma can create micro- and nanostructures on the surface of PTFE, which improve self-cleaning and hydrophobic properties [30,31]. The field of plasma surface engineering has made significant progress in recent years. The development of modern plasma reactors and atmospheric plasma systems has made the use of this technology on an industrial scale easier. Atmospheric pressure plasma systems are becoming particularly popular in the textile industry because they are less expensive and more easily used than vacuum systems [32]. With these systems, surface modification of textile materials can be incorporated into continuous industrial processes. The integration of nanotechnology with plasma engineering is also an important development. Nanoparticles can be deposited on textile surfaces by plasma treatment to create new functional properties (antibacterial properties or UV resistance). Similarly, the use of plasma polymerization in modern research is also increasing. This method can be used to form thin polymer films on textile surfaces that provide various functional properties. Furthermore, plasma treatment is also being used in combination with other surface modification techniques, e.g., sol–gel coating and chemical grafting, which further improve the performance of the materials [33].

Architectural textiles are becoming increasingly popular in the construction industry because they are lightweight, flexible and durable. These materials are used in building exteriors, facade systems, and membrane structures. Textile-based membrane structures are playing an important role in modern architecture because they have the ability to cover large areas with low weight. PTFE-coated fabrics are commonly used for this purpose because they are resistant to weathering and are long-lasting. Research has also shown that architectural textiles can help improve the energy efficiency of buildings. For example, sun-shading textile systems help control sunlight and heat, which can help balance the internal temperature of a building. Similarly, the use of textile materials is also increasing in modern facade systems. The use of textile membranes in double-layer facade systems helps to improve thermal insulation and reduce energy loss [34]. Recent studies have also shown that textile materials can be further improved by combining their performance with various functional coatings. For example, reflective coatings help to keep the internal temperature of a building low by reflecting the sun’s rays [35]. Textile polymer materials can be used in various retrofit systems of buildings due to their unique properties [36]. The use of textile-based membranes in thermal insulation systems helps in reducing energy loss. These membranes provide an additional protective layer on the outer surface of the building that reduces heat transfer [37]. Similarly, textile materials used in sun shading systems improve the internal environment of the building by controlling solar radiation. This results in reduced dependence on air conditioning systems and energy savings. Textile facade systems also play an important role in improving energy efficiency. These systems provide better thermal insulation to the outer structure of the building and protect it from environmental impacts. Textile materials can be made more energy efficient through advanced coatings and surface modifications. For example, highly reflective coatings help reduce the internal temperature of a building by reflecting solar heat.

A structured literature review methodology was adopted to evaluate the role of plasma-modified textile polymer materials in building energy retrofit applications. The review focused on studies investigating plasma-induced surface modification of polymer-based textile systems used in architectural membranes, facade retrofits, reflective roofing systems, smart shading structures, and thermal insulation applications. The analytical framework of this review was developed around the relationship between plasma surface engineering, polymer chemistry, and functional performance in energy-efficient building systems. Particular emphasis was placed on plasma-induced modifications affecting wettability, coating adhesion, thermal reflectivity, hydrophobicity, UV resistance, weather durability, and self-cleaning behavior. Comparative analysis was conducted across different polymer systems to evaluate how variations in chemical structure influence plasma responsiveness and retrofit performance. The selected textile polymer materials were specifically chosen due to their combination of lightweight structure, mechanical durability, environmental resistance, and compatibility with facade and membrane systems used in energy retrofit technologies. PET-based textiles are extensively used in architectural membranes and facade structures because of their high tensile strength, dimensional stability, and weather resistance. However, their relatively low surface energy and chemically inert surface can limit coating adhesion and wettability. Plasma treatment introduces oxygen-containing functional groups and controlled surface roughness, thereby improving coating compatibility and interfacial bonding performance [38]. PP-based textiles are widely applied in geotextiles, construction fabrics, and filtration systems because of their low density, chemical resistance, and cost-effectiveness. Nevertheless, the highly hydrophobic nature of PP restricts coating deposition and surface functionalization. Plasma activation enables the incorporation of polar functional groups onto the polymer surface, resulting in improved surface energy, wettability, and adhesion characteristics [39]. PTFE membranes are widely employed in tensile membrane architecture and advanced facade systems because of their exceptional chemical stability, UV resistance, and self-cleaning characteristics. Despite these advantages, PTFE exhibits extremely low surface reactivity, creating challenges for coating adhesion and interfacial modification [40]. Plasma treatment provides controlled surface activation and microstructural modification that enhance adhesion behavior and functional coating integration. PA fibers possess high mechanical strength, flexibility, and abrasion resistance, making them suitable for structural textile systems and technical applications. Plasma processing can further improve their surface activation behavior, enabling enhanced wettability, coating interaction, and multifunctional surface performance [41]. Glass fiber-reinforced textiles were included because of their importance in structural reinforcement systems and high-performance architectural composites [42]. These materials exhibit excellent thermal stability and mechanical durability; however, plasma treatment can significantly improve fiber–matrix interaction and coating compatibility through surface activation and chemical functionalization [43]. The reviewed studies were comparatively analyzed based on plasma treatment method, polymer type, induced surface modifications, characterization techniques, and building-energy-related performance indicators.

Critical evaluation of the reviewed studies indicates that plasma surface engineering provides a highly adaptable strategy for improving the multifunctional performance of textile polymer materials used in building energy retrofit systems. However, the effectiveness of plasma treatment is strongly dependent on the interaction between plasma parameters and polymer chemistry rather than on plasma exposure alone. One major inference from the literature is that plasma-induced performance enhancement is primarily governed by surface activation mechanisms involving oxidation, functional-group formation, and controlled micro/nano-scale roughening. These modifications improve coating adhesion, wettability control, and thermal functionality without significantly affecting the bulk mechanical properties of textile substrates. However, the magnitude of improvement varies substantially among different polymer systems because plasma responsiveness depends on polymer molecular structure, crystallinity, and surface energy characteristics. The reviewed studies also suggest that the most significant energy-related benefits are achieved when plasma treatment is combined with reflective or multifunctional coatings rather than used as an isolated surface modification process. Plasma treatment acts mainly as an enabling technology that improves coating compatibility, durability, and long-term functional stability of textile-based retrofit systems. Hydrophobicity and self-cleaning behavior are not only surface-functional properties but also indirectly contribute to long-term thermal efficiency. By reducing dust accumulation and maintaining higher solar reflectance over time, plasma-engineered surfaces help preserve the thermal-management performance of roofing and facade systems under outdoor environmental conditions.

## 3. Mechanism of Plasma–Polymer Surface Interaction

**Figure 2:**
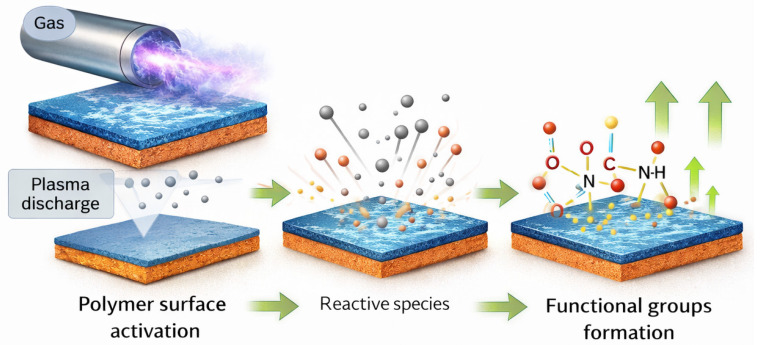
A schematic illustration of plasma–polymer surface interaction leading to functional group formation and enhanced surface properties.

The interaction between plasma and polymer surfaces involves a complex combination of physical and chemical processes that modify only the outermost surface region of the material without significantly affecting bulk properties. Plasma consists of a partially ionized gas containing energetic ions, electrons, radicals, neutral reactive species, metastable particles, and UV photons. When these reactive plasma species interact with textile polymer surfaces, they induce surface activation, bond scission, oxidation, etching, crosslinking, and micro/nano-scale morphological modification. The extent and nature of these modifications strongly depend on plasma operating conditions, gas composition, treatment duration, and polymer molecular structure. One of the initial effects of plasma exposure is surface cleaning [44]. Reactive plasma species remove organic contaminants, weak boundary layers, and low-molecular-weight surface impurities from the polymer surface through physical sputtering and chemical oxidation processes [45]. This cleaning effect increases surface purity and exposes chemically active sites, thereby improving coating adhesion and interfacial bonding performance. Another important plasma-induced phenomenon is the formation of chemically active functional groups [46]. During plasma treatment, energetic ions and radicals break polymer surface bonds and generate highly reactive free-radical sites. These active sites subsequently react with oxygen-, nitrogen-, or hydrogen-containing plasma species to form polar functional groups, including hydroxyl (–OH), carbonyl (C=O), carboxyl (–COOH), and amine-containing functionalities. The incorporation of these groups significantly increases surface energy and improves wettability, coating adhesion, and compatibility with functional surface layers. This mechanism is particularly important for hydrophobic polymers (PP and PTFE), which naturally exhibit low surface energy and poor adhesion behavior. Plasma treatment also induces controlled surface roughening and micro/nano-scale texturing through plasma etching mechanisms [47]. Energetic plasma particles selectively remove fragments of polymer chains from the surface, generating microstructures and nanoscale roughness. These topographical changes increase the effective surface area and alter liquid-surface interaction behavior. In textile polymers, plasma-induced roughness contributes to improved mechanical interlocking of coatings and enhanced hydrophobic or self-cleaning performance depending on the resulting surface chemistry. Crosslinking is another important process occurring during plasma exposure, particularly in oxygen-deficient or low-energy plasma environments. In this mechanism, fragmented polymer chains recombine to form interconnected surface networks, resulting in improved chemical stability, abrasion resistance, and environmental durability. However, excessive plasma exposure may also cause aggressive surface degradation, chain scission, or over-etching, which can negatively affect the mechanical integrity of sensitive textile fibers [48]. Therefore, optimization of plasma parameters is critical to achieving controlled surface functionalization without damaging the substrate material. The plasma–polymer interaction mechanism varies considerably among different textile polymers because of differences in molecular structure, crystallinity, bond dissociation energy, and surface chemistry. Plasma surface engineering provides a highly versatile and surface-selective approach for tailoring the physicochemical properties of textile polymers. The mechanism of plasma–polymer surface interaction is presented in Figure 2.

Plasma processing of textile polymer materials differs significantly from plasma treatment of conventional flat polymer films or bulk materials because textile substrates possess complex fibrous architectures, porous structures, high surface-area-to-volume ratios, and mechanically flexible geometries. These characteristics strongly influence plasma–surface interaction behavior, treatment uniformity, gas diffusion, surface activation efficiency, and long-term functional performance. One of the most important features of textile materials is their hierarchical structure consisting of fibers, yarns, and interconnected porous networks [49,50]. Unlike smooth polymer films, textile substrates contain irregular topography and internal void spaces that affect plasma penetration and reactive-species distribution. As a result, plasma exposure may not occur uniformly across the entire textile structure. Surface fibers are generally exposed more directly to reactive plasma species, whereas internal fibers located within densely woven or multilayer textile systems may experience lower plasma intensity. This can lead to heterogeneous surface modification and non-uniform coating adhesion behavior. Therefore, a comprehensive characterization of plasma-treated textile polymer surfaces is essential for understanding the relationship between plasma-induced surface modification and functional performance in building energy retrofit applications. Plasma treatment primarily alters the outermost surface layer of polymer materials through chemical activation, micro/nano-scale roughening, etching, and functional-group incorporation. Therefore, multiple complementary analytical techniques are required to evaluate morphological, chemical, and wettability-related changes induced by plasma exposure. Scanning Electron Microscopy (SEM) is widely used to examine plasma-induced morphological modifications on textile polymer surfaces. SEM analysis provides high-resolution visualization of fiber topography, surface etching, micro-roughness formation, and structural changes resulting from plasma interaction [51]. These morphological changes are directly associated with improved coating adhesion, increased surface area, and enhanced interfacial bonding between textile substrates and functional coatings. In architectural membrane and facade applications, SEM observations help assess the uniformity and effectiveness of plasma treatment, particularly for reflective coatings and protective surface layers exposed to outdoor environmental conditions. Atomic Force Microscopy (AFM) enables quantitative nanoscale characterization of surface roughness and topographical features generated during plasma treatment. Unlike SEM, AFM provides three-dimensional (3D) surface profiling and roughness measurements at nanometer resolution, allowing precise evaluation of plasma-induced nano-structuring effects [52]. Increased nanoscale roughness strongly influences wettability behavior, hydrophobicity, and self-cleaning performance by modifying liquid-surface interaction mechanisms. These properties are particularly relevant for reflective roofing systems and smart facade textiles, where maintaining clean and highly reflective surfaces is critical for long-term thermal management and energy efficiency.

**Figure 3:**
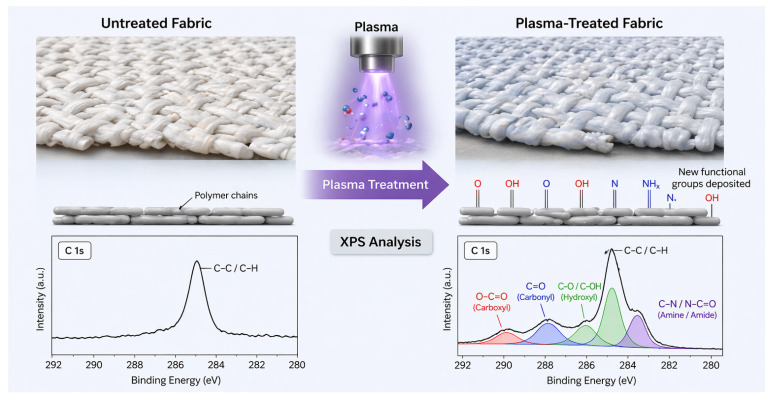
The image shows the plasma-treated surface (with air and nitrogen gas) of PET. On untreated surface, only hydrocarbon (C−C/C−H) peak is observed. There are no oxygen- and nitrogen-containing groups. While on treated plasma surface, new peaks for O− and N− containing groups are observed, (−COOH, −OH, −C=OH, −C−N), indicating deposition of functional groups.

Fourier Transform Infrared Spectroscopy (FTIR) is employed to identify chemical functional groups formed on polymer surfaces after plasma exposure. Plasma treatment introduces oxygen- and nitrogen-containing groups, which significantly alter surface chemistry and increase surface energy [53]. FTIR analysis therefore provides important evidence of plasma-induced chemical activation and oxidation mechanisms. The formation of these functional groups is directly linked to improved wettability, enhanced coating adhesion, and increased compatibility between textile substrates and protective or reflective coatings used in retrofit systems. X-ray Photoelectron Spectroscopy (XPS) is one of the most important surface-sensitive techniques for evaluating the elemental composition and chemical-state changes in plasma-treated polymers. XPS enables quantitative analysis of surface oxidation, functional-group incorporation, and plasma-induced chemical modification within the outermost nanometer-scale surface region [54]. This technique is particularly valuable for confirming the successful incorporation of oxygen- or nitrogen-containing species responsible for enhanced adhesion and durability. In building-envelope applications, XPS analysis helps evaluate the long-term chemical stability and environmental resistance of plasma-engineered textile surfaces. Contact-angle measurement is commonly used to evaluate changes in surface wettability after plasma treatment. Variations in water contact angle provide indirect assessment of surface energy, hydrophilicity, hydrophobicity, and self-cleaning behavior [55]. The visualization of plasma treatment and XPS analysis (presence of functional groups) is presented in Figure 3. Plasma-induced reductions or increases in contact angle are closely associated with changes in surface chemistry and roughness. In building retrofit applications, wettability behavior is highly important because it influences water repellency, dust accumulation, coating stability, and long-term reflective performance of roofing and facade materials. Consequently, contact-angle analysis serves as an important indicator of the functional efficiency and environmental durability of plasma-modified textile systems.

In addition to surface chemical and morphological characterization, evaluation of the mechanical and thermomechanical behavior of plasma-modified textile polymer materials is essential for understanding their long-term structural performance in building-envelope and architectural applications [56,57]. Textile materials used in facade systems, tensile membrane structures, reflective roofing assemblies, and multilayer retrofit systems are continuously subjected to mechanical loading, thermal cycling, environmental aging, and interfacial stress conditions [58,59]. Consequently, plasma-induced surface modification must be analyzed not only from a surface-science perspective, but also in terms of continuum mechanics, structural durability, and thermomechanical stability. Tensile testing is one of the most important characterization methods for evaluating the mechanical integrity of plasma-treated textile systems [60]. Plasma exposure may influence fiber-fiber interaction, surface cohesion, coating adhesion, and interfacial bonding behavior, which can subsequently affect tensile strength, elongation behavior, stiffness, and fracture resistance. In optimized plasma-treatment conditions, bulk mechanical properties are generally preserved because plasma modification primarily affects the outermost surface region. Fatigue and creep behavior are additional important considerations for plasma-engineered textile systems used under sustained or cyclic loading conditions [61]. Architectural membranes and tensile textile structures experience continuous wind loading, thermal expansion, moisture cycling, and mechanical deformation throughout their service life. Plasma-modified coating interfaces and surface-functionalized fibers must therefore maintain structural integrity under repetitive stress conditions. Long-term creep analysis and cyclic mechanical testing are particularly important for evaluating interfacial durability and coating stability in multilayer textile systems.

## 4. Plasma-Induced Surface Structural Modification

**Figure 4:**
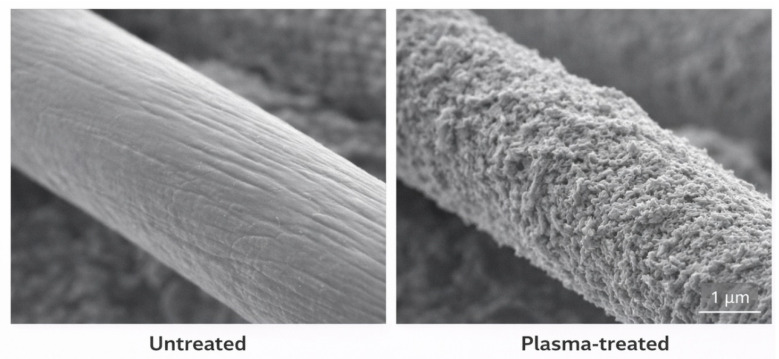
SEM images showing surface morphology of textile fibers before and after plasma treatment. Reprinted with permission [62].

Plasma surface engineering induces substantial physicochemical modifications in textile polymer materials by altering the outermost surface region through energetic plasma–surface interactions. Reactive plasma species (ions, radicals, electrons, metastable particles, and UV photons) interact with polymer chains and initiate oxidation, bond scission, etching, crosslinking, and surface restructuring processes. One of the most prominent effects of plasma treatment is the development of surface roughness and hierarchical micro/nano-scale structures. Plasma etching selectively removes low-molecular-weight surface fragments and weak boundary layers, generating controlled topographical modification on textile fibers. SEM investigations (Figure 4) performed by Sun and Stylios on oxygen plasma-treated PET fabric revealed the formation of micro-scale surface irregularities and fiber roughening after plasma exposure [62]. Similarly, DBD plasma treatment of PP fibers produced nanoscale surface texturing and increased morphological heterogeneity, resulting in enhanced water interaction behavior and improved surface activation [63]. The extent of roughness development depends strongly on plasma operating parameters and polymer chemistry. Hydrophobic polymers generally exhibit more pronounced etching and surface texturing because of their low surface energy and relatively inert surface characteristics. In contrast, PET materials often demonstrate stronger oxidation behavior and functional-group incorporation. Plasma-induced roughening increases effective surface area and promotes mechanical interlocking between textile substrates and functional coatings, thereby improving coating adhesion and long-term interfacial stability. In addition to morphological modification, plasma treatment significantly alters surface chemistry through the incorporation of reactive functional groups. Energetic plasma species break polymer surface bonds and generate highly reactive radical sites, which subsequently react with oxygen- or nitrogen-containing plasma species to form hydroxyl, carbonyl, carboxyl, and amine functionalities. Friedrich reported that plasma-induced chain scission and surface oxidation fundamentally alter polymer surface chemistry and interfacial properties [64,65].

The incorporation of polar functional groups increases surface energy and improves wettability, coating compatibility, and adhesion performance. These effects are particularly important for textile-based facade systems and reflective roofing membranes where strong coating adhesion and environmental durability are essential for long-term thermal efficiency. Plasma treatment further promotes the formation of hierarchical micro-scale and nano-scale surface architectures that strongly influence liquid-surface interaction behavior. AFM investigations revealed significant nanoscale roughness development after plasma exposure, confirming the formation of nano-textured surface domains on textile fibers [66]. These nano-structured surfaces can alter wetting behavior according to Wenzel and Cassie-Baxter wetting mechanisms, depending on the resulting surface chemistry and roughness distribution. Increased surface roughness combined with low-surface-energy functionalization may produce hydrophobic or superhydrophobic behavior characterized by reduced liquid contact area and enhanced self-cleaning performance. Surface-energy evolution is another critical consequence of plasma treatment. Contact-angle investigations by Strobel and Lyons demonstrated substantial increases in surface energy following plasma exposure due to surface oxidation and functional-group incorporation [67]. Improved surface energy facilitates uniform spreading of coatings and enhances interfacial bonding performance. However, excessive plasma exposure may also induce over-etching or surface degradation, which can negatively affect fiber integrity and coating durability. Therefore, optimization of plasma-treatment parameters is essential for balancing surface activation and structural preservation. The reviewed studies further indicate that plasma-induced modifications vary significantly among different polymer systems because of differences in molecular structure, crystallinity, bond dissociation energy, and fluorination level. PET generally exhibits strong oxidation and enhanced wettability after oxygen plasma treatment, whereas PTFE demonstrates more pronounced surface etching and microstructural modification because of its chemically inert surface characteristics [68]. PP, on the other hand, displays substantial improvements in wettability and coating adhesion after plasma-induced incorporation of polar functional groups.

## 5. Functional Surfaces for Thermal Management and Environmental Durability

One of the most important advantages of plasma surface engineering is its ability to simultaneously modify multiple functional properties of textile polymer materials relevant to building energy retrofit systems. Plasma-induced surface activation, roughness evolution, and chemical functionalization collectively influence wettability, self-cleaning behavior, solar reflectance, coating stability, and environmental durability [69]. These properties are strongly interconnected in architectural textile applications because the long-term thermal performance of building-envelope materials depends not only on their initial reflectivity or insulation capability, but also on their ability to resist contamination, moisture accumulation, UV degradation, and environmental weathering. In building-envelope systems (reflective roofing membranes, facade textiles, and smart shading structures), surface contamination caused by dust, pollutants, and atmospheric particles can significantly reduce solar reflectance and thermal-regulation efficiency over time [70]. Consequently, plasma-engineered hydrophobic and self-cleaning surfaces are not only surface-functional modifications but also important mechanisms for preserving long-term energy performance. Similarly, plasma-enhanced coating adhesion improves the durability and operational stability of reflective and protective surface layers under outdoor environmental exposure. A hydrophobic surface is one on which water droplets form a sphere instead of spreading. This property is usually assessed by contact angle measurement. If the contact angle of a water droplet is greater than 90°, the surface is considered hydrophobic, while if it is greater than 150°, the surface is called superhydrophobic [71]. In a study, Briga-Sá et al. reported that self-cleaning surfaces are very useful for building facade systems because they keep the surface clean for a long time [72].

Thermal reflectivity is an important factor in improving the energy efficiency of buildings. The roofs and external walls of buildings directly absorb solar radiation, raising internal temperatures and increasing reliance on cooling systems. Solar reflectivity is the ability of a surface to reflect back the incoming solar energy instead of absorbing it. If the reflectivity of a surface is high, it absorbs less heat and as a result, the internal temperature of the building decreases. Plasma treatment is often used in combination with reflective coatings. Reflective coatings typically consist of metallic or ceramic particles that are capable of reflecting solar radiation. However, these coatings require effective adhesion. In a study, Synnefa et al. studied the energy efficiency of buildings using reflective coatings and reported that surfaces with high solar reflectance can reduce the temperature of the building by several degrees. When these same coatings are applied to plasma-modified textile surfaces, their performance can be further improved [73]. In another study, PET was treated with oxygen plasma and then coated with a reflective coating. Experimental results showed that the uniformity of the coating improved after plasma treatment and an increase in solar reflectance of about 10–15% was observed [74]. During plasma treatment, micro- and nano-surface structures are also formed. This structure improves the mechanical interlocking between the coating and the base material. It was reported that after plasma treatment, fine roughness is formed on the fiber surface, which provides a better base for the coating [75]. The effects of plasma treatment may vary between different polymer materials because each polymer has a different chemical structure. The surface energy of PET increases significantly after plasma treatment, which improves the adhesion of various coating materials (polyurethane and acrylic) [76]. The surface of PP is hydrophobic, which makes it difficult to apply coatings to it. Polar groups can be created on its surface by plasma treatment, which improves coating adhesion [77]. PTFE is considered a difficult material for coating applications due to its low surface energy. Its surface can be functionalized by plasma treatment to improve coating adhesion [78].

Polymer materials generally undergo chemical changes under the influence of UV rays. When UV rays affect polymer molecules, the chemical bonds in them can be broken, which results in the process of photooxidation. This process can affect the mechanical strength, elasticity and surface properties of the polymer [79]. According to Jelle, UV stability is a fundamental requirement for materials used in the exterior of buildings, as prolonged exposure to sunlight can weaken the structure of the material [80]. Plasma treatment induces various chemical changes on the polymer surface that can improve the resistance of the material to UV radiation. Cross-linking can be increased on the polymer surface during plasma treatment, which makes the molecular structure more stable and slows down UV degradation. PP is relatively sensitive to UV radiation because its molecular structure is susceptible to photooxidation. Plasma treatment makes it easier to apply protective coatings to its surface, which improves the durability of the material [81].

Exterior materials of buildings are exposed not only to UV radiation but also to factors such as precipitation, humidity and temperature changes. The weathering resistance of materials can be increased by improving the surface properties [82]. Several experimental studies have shown that the weathering resistance of textile materials improves after plasma treatment [83,84]. The performance of the protective coating applied to PET after plasma treatment was maintained for a long time and the mechanical properties of the material were observed to be less degraded even after UV exposure. In a study, accelerated weathering tests were performed on PP fibers after plasma treatment, which showed that the surface structure of the material remained more stable after plasma treatment. Durability of materials is of utmost importance in architectural textile systems, as these structures are directly exposed to the environment. It is important to design architectural textile materials in such a way that they can withstand weathering effects for a long time [85]. The materials used in the building energy retrofit process must remain effective for a long time so that the energy-saving benefits can be maintained. If the textile materials deteriorate quickly, the performance of the retrofit systems may be affected. The reviewed studies indicate that hydrophobicity, self-cleaning behavior, and thermal reflectivity should not be considered independent functional properties in textile-based building retrofit systems. Instead, these characteristics interact synergistically to preserve long-term thermal-management performance under environmental exposure conditions. This synergistic interaction between wettability control, contamination resistance, and thermal reflectivity is particularly important for architectural membranes and roofing systems deployed in polluted urban environments where long-term performance degradation remains a major challenge.

## 6. Quantitative Link Between Plasma Surface Modification and Building Energy Savings

The contribution of plasma-modified textile polymer materials to building energy savings can be understood through the relationship between surface modification, thermal management, and cooling-load reduction. Plasma treatment does not directly reduce building energy consumption by itself; rather, it improves the surface characteristics of textile polymers so that reflective, protective, and functional coatings can perform more effectively in building envelope applications. The reviewed studies indicate that plasma treatment increases surface roughness, introduces polar functional groups, and improves coating adhesion on textile polymer substrates [86,87]. These changes enhance the uniformity and durability of reflective coatings applied to roofing textiles, facade membranes, and shading systems. Plasma-treated textile surfaces showed water contact angle changes from approximately 70° to 145°, depending on polymer type and plasma conditions, indicating substantial modification of surface wettability and hydrophobic performance [88,89]. Improved hydrophobicity and self-cleaning behavior are important for maintaining solar reflectance because dust accumulation can reduce the reflective efficiency of roofing and facade materials. Quantitative findings reported in the reviewed literature further support this connection. Plasma-treated textile roofing systems combined with reflective coatings showed approximately 10–15% improvement in solar reflectance [90]. In experimental roofing applications, reflective plasma-treated textile materials contributed to roof surface temperature reductions of approximately 10–15 °C [91]. Lower roof surface temperature reduces conductive heat transfer into the building interior, thereby decreasing cooling load. Similarly, plasma-treated smart shading textiles were reported to reflect approximately 60% of incident solar radiation, resulting in indoor temperature reductions of approximately 3–4 °C and air-conditioning energy savings of approximately 18–25% [92]. These findings demonstrate that the energy-saving potential of plasma-modified textile polymers is mainly indirect but technically significant. Plasma treatment enhances surface activation, coating adhesion, hydrophobicity, and durability. These improvements help maintain high solar reflectance and thermal-control performance under outdoor exposure. Consequently, plasma-modified textile systems can contribute to reduced solar heat gain, lower cooling demand, and improved long-term energy performance of building envelopes.

## 7. Textile Formation Technologies and Their Influence on Plasma Surface Engineering

The structural formation technology of textile polymer materials plays a critical role in determining plasma-treatment behavior, coating interaction, mechanical performance, and long-term functionality in building energy retrofit applications [93,94]. Unlike conventional polymer films, textile materials possess complex hierarchical architectures formed through fiber production, yarn assembly, fabric construction, and multilayer composite processing [95]. These structural characteristics strongly influence plasma penetration, surface activation efficiency, wettability behavior, thermal transport, and durability performance. Textile polymer materials used in architectural and retrofit systems are commonly manufactured through weaving, knitting, nonwoven processing, lamination, coating, and composite reinforcement technologies [96]. Each fabrication approach produces distinct surface morphology, porosity, fiber orientation, and mechanical behavior, which subsequently affect plasma–surface interaction mechanisms. Woven textile structures are widely used in architectural membranes and facade systems because of their high dimensional stability, tensile strength, and structural durability [97]. However, tightly woven structures may restrict penetration of reactive plasma species into internal fiber regions, potentially resulting in non-uniform surface activation. In contrast, knitted textile systems possess greater flexibility and porosity, which can improve plasma accessibility but may also introduce challenges related to dimensional stability and coating uniformity. Nonwoven textile materials provide high surface area and interconnected porous structures that facilitate plasma penetration and functional-group incorporation. These materials are particularly attractive for filtration systems, insulation layers, and multilayer retrofit composites. However, their irregular fiber arrangement can lead to heterogeneous plasma exposure and variable surface modification behavior. Electrospun nanofiber systems represent another emerging class of textile polymer materials with significant potential for plasma-assisted functionalization. Because electrospun structures possess extremely high specific surface area and nanoscale fiber morphology, plasma treatment can efficiently modify surface chemistry and wettability. Plasma-engineered electrospun textiles are increasingly being investigated for smart membranes, multifunctional coatings, and adaptive thermal-management systems [98].

In architectural textile applications, polymer fibers are often combined with coating and lamination technologies to produce high-performance membrane systems. PTFE-coated glass fibers and PET membranes are common examples used in tensile structures and reflective facade systems [99]. Plasma treatment is frequently applied as a pre-treatment step to improve coating adhesion, interfacial bonding, and long-term durability of these multilayer assemblies. The formation technology also strongly influences the mechanical and environmental behavior of textile materials under operational conditions. Fiber orientation, weave density, porosity, and reinforcement architecture affect tensile response, moisture transport, thermal conductivity, and resistance to environmental loading [100,101,102]. Consequently, plasma-processing parameters must be optimized according to the specific textile structure to avoid excessive etching, fiber degradation, or loss of mechanical integrity. From a building-energy perspective, textile architecture directly affects thermal-management behavior, solar–radiation interaction, air permeability, and moisture regulation. Therefore, the relationship between textile formation technology and plasma-induced surface modification is essential for designing multifunctional textile systems with improved thermal reflectivity, self-cleaning performance, durability, and energy efficiency.

## 8. Case Studies

To understand the practical application of plasma-modified textile polymer materials, it is important to review various research and industrial case studies. Two important case studies in which plasma-treated textile materials were used to improve the energy performance of buildings are presented below:

### 8.1. Case Study 1: Reflective Plasma-Treated Roofing Textiles for Cooling Energy Reduction

Building roofs are a major source of solar heat gain. Especially in hot climates, roof heat gain increases the internal temperature of the building, which results in increased reliance on cooling systems. To address this problem, reflective roofing systems are used that reflect solar radiation. In a research study, PET fabric was treated with oxygen plasma and then a reflective ceramic coating was applied to it [103]. After plasma treatment, contact angle measurements and surface energy analysis showed that the surface energy increased significantly, which allowed the coating to spread better over the surface. When this material was applied to the roof of an experimental building, an increase in solar reflectance of approximately 12–18% was observed. The use of reflective coatings can reduce roof temperatures by 10–15 °C. The experiments conducted in this case study also showed that the coating maintained its performance for a long time due to improved adhesion of the coating after plasma treatment. Energy analysis showed that the use of reflective plasma-treated roofing textiles reduced the cooling energy consumption of the building by approximately 20% [104]. This case study shows that energy savings can be achieved by using plasma-modified textile materials in reflective roofing systems.

### 8.2. Case Study 2: Smart Plasma-Treated Textile Shading Systems for Energy-Efficient Buildings

The use of shading systems is considered to be very effective for solar heat control in buildings. If adequate shading is provided on windows and facades, solar heat can be prevented from entering the building interior. Appropriate shading systems can reduce the cooling energy consumption of buildings by about 30% [105]. In a research project, atmospheric plasma treatment was applied to PP fabric to improve its surface properties. After plasma treatment, an increase in surface roughness and wettability was observed, which allowed for the effective deposition of reflective nanoparticles on the surface [106]. The textile was then installed in an experimental building as a smart shading system. Experimental results showed that the plasma-treated shading textile was able to reflect about 60% of the solar radiation. As a result, an average decrease of 3–4 °C was observed in the internal temperature of the building. Furthermore, energy analysis showed that the use of this system reduced the energy consumption of air conditioning by about 18–25%. Self-cleaning properties were also observed in this case study as the textile surface became more hydrophobic after plasma treatment.

## 9. Industrial and Technical Challenges

Despite the promising laboratory-scale performance of plasma-modified textile polymer materials, several practical barriers continue to limit large-scale industrial implementation in building energy retrofit systems. One of the most significant challenges is maintaining treatment uniformity during continuous processing of large textile surfaces. Architectural membranes and facade textiles are commonly manufactured in roll widths exceeding several meters, whereas many plasma studies are performed on relatively small laboratory specimens. Non-uniform plasma exposure across wide textile surfaces can lead to inconsistent surface activation, variable coating adhesion, and non-uniform wettability behavior, ultimately affecting long-term functional performance. Atmospheric-pressure plasma and DBD systems are currently considered the most industrially viable plasma technologies because they can be integrated into continuous roll-to-roll textile processing lines [107]. However, industrial implementation requires precise control of plasma power density, treatment speed, gas composition, electrode configuration, and substrate positioning. In high-throughput manufacturing environments, maintaining stable plasma conditions while achieving reproducible surface functionalization remains technically challenging. Another important implementation issue is the long-term stability of plasma-induced surface modification under real environmental conditions. Many laboratory investigations evaluate plasma-treated materials immediately after treatment; however, architectural textiles used in roofing and facade systems are exposed to UV radiation, humidity fluctuations, temperature cycling, wind-driven particles, and atmospheric contaminants for extended periods. Under these conditions, plasma-induced functional groups may gradually rearrange or degrade, resulting in hydrophobic recovery and partial loss of surface activation [108]. This phenomenon may reduce coating adhesion and compromise the long-term reflective or self-cleaning performance of textile retrofit systems.

Practical implementation also depends strongly on compatibility between plasma treatment and industrial coating technologies. In reflective roofing membranes and facade textiles, plasma treatment is often used as a pre-treatment step before deposition of ceramic, fluoropolymer, or nanoparticle-based coatings. Although plasma activation improves coating adhesion and spreading behavior, industrial coating lines require synchronization between plasma treatment and coating deposition because plasma-induced surface activation may decay over time. Delays between treatment and coating application can therefore reduce process effectiveness in large-scale manufacturing. Economic feasibility remains another major challenge for commercialization. Although plasma processing reduces the use of liquid chemicals and solvent-based surface treatments, the capital cost of plasma reactors, power systems, gas-delivery infrastructure, and process-control equipment can be significant. Furthermore, operational costs associated with energy consumption, maintenance, electrode degradation, and process monitoring may affect economic competitiveness compared with conventional textile finishing technologies. At present, comprehensive techno-economic assessments and lifecycle cost analyses for plasma-modified textile retrofit systems remain limited in the literature. Additional challenges include fire-safety compliance, environmental durability certification, weathering resistance validation, and compatibility with existing construction standards. Building-envelope materials must satisfy strict regulatory requirements related to flame resistance, mechanical durability, moisture stability, and long-term environmental exposure.

## 10. Economic and Life Cycle Assessment (LCA) Considerations

Although plasma treatment offers significant functional advantages, its economic feasibility and environmental sustainability remain important considerations for large-scale implementation in building energy retrofit systems. Compared with conventional wet-chemical surface treatment methods, plasma processing is generally regarded as a cleaner and more environmentally favorable technology because it minimizes the use of liquid chemicals, solvents, and water-intensive finishing operations [109]. Plasma treatment also reduces the generation of hazardous chemical waste and secondary effluents commonly associated with traditional textile surface modification processes. Atmospheric-pressure plasma and DBD systems are considered more economically attractive for industrial textile processing because they can be integrated into continuous roll-to-roll manufacturing lines without vacuum chambers. However, energy consumption, electrode maintenance, gas utilization, and process monitoring remain important cost-related factors influencing commercial scalability. Plasma-modified textile systems may contribute to sustainability through improved material durability, enhanced coating adhesion, and prolonged functional performance. Increased resistance to environmental degradation, contamination, and coating delamination can extend the operational lifespan of roofing membranes, facade textiles, and reflective building-envelope systems, thereby reducing maintenance frequency and material replacement demand. In addition, plasma-engineered hydrophobic and self-cleaning surfaces may reduce cleaning-related water consumption and chemical usage during long-term building operation. The reviewed literature further suggests that plasma-enhanced reflective textile systems can contribute indirectly to lifecycle energy savings by reducing building cooling demand and mitigating solar heat gain. Improved solar reflectance and thermal regulation may lower operational energy consumption over the service life of building-envelope materials. However, the overall environmental benefit depends on balancing these operational energy savings against the energy required for plasma processing and coating deposition during manufacturing. At present, comprehensive LCA studies focused specifically on plasma-modified textile polymer systems for building retrofit applications remain limited. Most available studies emphasize immediate functional-property enhancement rather than cradle-to-grave environmental assessment. Important factors, e.g., embodied energy, carbon footprint, recyclability, end-of-life treatment, and long-term environmental impact, have not yet been systematically investigated for plasma-engineered architectural textile systems.

## 11. Existing Limitations

Although plasma surface engineering has demonstrated significant potential for textile polymer materials, the current literature still presents several scientific and technological limitations that restrict large-scale implementation in building energy retrofit systems. One of the major limitations in existing studies is the lack of standardized plasma treatment conditions. Reported experimental parameters (plasma power, treatment duration, gas composition, discharge configuration, and operating pressure) vary considerably among studies, making direct comparison of results difficult [110]. Consequently, reported improvements in wettability, coating adhesion, and thermal reflectivity often differ substantially even for the same polymer material. Plasma-treated PET fabric has shown varying contact-angle behavior depending on plasma gas type and exposure time, indicating that plasma-induced surface modification is highly process-dependent rather than universally reproducible. Another important limitation is the insufficient investigation of long-term durability and surface aging effects. Many studies report immediate improvements in hydrophobicity, adhesion, or surface activation after plasma treatment; however, relatively few investigations evaluate the stability of these properties under prolonged environmental exposure. Plasma-induced functional groups may undergo molecular rearrangement over time, leading to partial recovery of hydrophobicity and reduction in surface energy [111]. This issue is particularly critical for architectural and facade applications where materials are exposed to UV radiation, humidity, temperature cycling, and atmospheric pollutants for extended periods. The literature also remains heavily focused on laboratory-scale experimentation, while comparatively limited work addresses industrial scalability and real-building implementation. Although atmospheric-pressure plasma and DBD systems are considered promising for continuous textile processing, challenges related to treatment uniformity, energy consumption, processing speed, and operational cost remain insufficiently addressed.

Another critical issue is the limited availability of direct building-energy-performance validation. Many studies infer energy-saving potential from improved reflectivity, hydrophobicity, or thermal properties, but relatively few investigations provide comprehensive building-scale thermal simulations or long-term field measurements. As a result, the relationship between plasma-induced surface modification and actual reductions in building energy consumption remains partially indirect in many cases. More integrated studies combining materials engineering, thermal modeling, and real environmental testing are needed to establish stronger quantitative links between plasma-treated textile systems and building energy performance. In addition, existing research primarily focuses on single-function surface improvements (hydrophobicity or coating adhesion), whereas future building materials will likely require multifunctional performance including thermal management, self-cleaning behavior, UV resistance, mechanical durability, and compatibility with renewable-energy systems. The integration of plasma engineering with nanotechnology, smart coatings, and adaptive textile systems remains an emerging research area requiring deeper interdisciplinary investigation.

## 12. Future Perspectives and Research Opportunities

Plasma-modified textile polymer materials have shown significant potential in the field of building energy retrofit. These materials have opened up new ways to improve the energy efficiency of buildings, reduce heat loss and control the effects of solar heat gain. However, research in this area is still in its early stages, and there is great potential for further development in the future. The potential for using plasma-modified textile materials, especially in architectural and building retrofit systems, has not yet been fully explored. Therefore, further research on this topic is needed to better understand the practical performance, long-term sustainability, and economic viability of these materials. Plasma surface engineering can be combined with nanotechnology to achieve new functional properties. Nanoparticles can be deposited on textile surfaces by plasma treatment, which can significantly improve the performance of the material. Plasma surface modification can be used to create platforms on which various smart coatings or sensors can be installed. This can lead to the development of textile systems that can adjust their properties according to temperature, humidity or solar radiation. In the future, textile polymer materials can also be integrated with renewable energy systems.

Although significant progress has been achieved in experimental plasma surface engineering of textile polymer materials, the integration of computational modeling and digital simulation into this field remains comparatively limited. Modeling of plasma treatment processes is important because plasma-induced surface modification depends on multiple coupled parameters including plasma power, gas composition, pressure, treatment duration, discharge configuration, and substrate geometry. Numerical approaches, e.g., computational fluid dynamics, plasma kinetics modeling, and finite-element simulation, can provide valuable insight into plasma-species distribution, energy transfer mechanisms, ion bombardment behavior, and surface-reaction dynamics. Such models may help optimize plasma-treatment conditions for textile substrates while minimizing excessive etching, thermal degradation, and non-uniform surface activation. The modeling of textile behavior under mechanical and environmental loading conditions is particularly important for architectural and building-envelope applications. Plasma-modified textile systems used in facade membranes, tensile structures, and reflective roofing applications are continuously exposed to wind loading, thermal cycling, UV radiation, humidity variation, and particulate contamination. These loading conditions can influence coating stability, fiber degradation, surface aging, and long-term thermal performance. Finite-element modeling and structural simulation can therefore provide important insight into stress distribution, deformation behavior, fatigue resistance, and durability of plasma-engineered textile systems under real operating conditions.

Digital technologies, e.g., artificial intelligence (AI), machine learning, and digital twin systems, also offer promising opportunities for future plasma-engineering research. These tools are already showing their potential in various scientific fields, including advanced materials research [112,113]. Data-driven optimization models could help establish relationships between plasma-processing parameters, surface morphology evolution, wettability behavior, and energy-performance characteristics. Similarly, AI-assisted predictive frameworks may accelerate the design of multifunctional textile surfaces with tailored thermal, hydrophobic, and durability properties for specific environmental conditions. This can lead to more efficient and energy-efficient plasma processes [114]. Although there is a lot of research on the immediate effects of plasma treatment, more research is still needed on long-term performance. 

## 13. Conclusions

This review critically evaluated the potential of plasma-modified textile polymer materials for advanced building energy retrofit applications by integrating findings from plasma surface engineering, polymer science, and architectural textile research. The reviewed literature demonstrates that plasma treatment is an effective surface-functionalization strategy capable of significantly modifying the physicochemical properties of textile polymers without substantially altering their bulk mechanical characteristics. Plasma-induced oxidation, surface activation, controlled etching, and micro/nano-scale roughening collectively improve wettability, coating adhesion, hydrophobicity, thermal reflectivity, UV resistance, and environmental durability of textile-based building materials. Key findings are given below:

- Among the investigated plasma technologies, atmospheric-pressure plasma and DBD systems appear particularly promising for large-scale textile processing because of their compatibility with continuous industrial manufacturing and relatively low thermal impact on polymer substrates. PET, PP, PTFE, and fiber-reinforced composites each exhibit different plasma-response mechanisms depending on their molecular structure and surface chemistry.
- The literature further suggests that plasma-engineered textile systems can contribute indirectly but significantly to building energy savings through improved solar reflectance, enhanced thermal management, and prolonged functional durability of roofing, facade, and shading materials. However, despite considerable progress at the laboratory scale, several critical challenges continue to limit broader implementation. These include insufficient standardization of plasma treatment parameters, limited long-term environmental durability studies, plasma aging effects, scalability constraints, inconsistent performance reporting, and the lack of comprehensive building-scale thermal validation under real operating conditions.
- A major research gap identified in the current literature is the absence of integrated multidisciplinary studies combining plasma engineering, thermal simulation, durability assessment, and real-building energy analysis. Most existing investigations focus primarily on immediate surface-property modification rather than long-term operational performance and lifecycle effectiveness. Furthermore, relatively limited attention has been given to techno-economic analysis, environmental impact assessment, and industrial feasibility of plasma-assisted textile retrofit technologies.
- Future research should therefore prioritize the development of standardized plasma-treatment protocols for textile polymers; long-term outdoor weathering and durability evaluation; integration of plasma-functionalized textiles with smart coatings and adaptive facade systems; coupling of plasma-modified surfaces with renewable-energy technologies (photovoltaic membranes); and data-driven optimization of plasma processing using digital modeling approaches.
- Plasma-modified textile polymer materials represent a highly promising class of multifunctional building-envelope materials for sustainable retrofit technologies. Their ability to combine lightweight structural performance with tunable surface functionality offers significant opportunities for the development of next-generation energy-efficient architectural systems.

## Data Availability

The original contributions presented in this study are included in the article. Further inquiries can be directed to the corresponding authors.
